# Cationic nanocarriers as potent adjuvants for recombinant S-RBD vaccine of SARS-CoV-2

**DOI:** 10.1038/s41392-020-00434-x

**Published:** 2020-12-11

**Authors:** Hong Lei, Aqu Alu, Jingyun Yang, Cai He, Weiqi Hong, Zesheng Cheng, Li Yang, Jiong Li, Zhenling Wang, Wei Wang, Guangwen Lu, Xiawei Wei

**Affiliations:** 1 Laboratory of Aging Research and Cancer Drug Target, State Key Laboratory of Biotherapy and Cancer Center, National Clinical Research Center for Geriatrics, West China Hospital, Sichuan University, 610041 Sichuan, Chengdu China; 2 Emergency Department, State Key Laboratory of Biotherapy, West China Hospital, Sichuan University, 610041 Sichuan, Chengdu China

**Keywords:** Vaccines, Infectious diseases

**Dear Editor**,

The worldwide outbreak of severe acute respiratory syndrome coronavirus 2 (SARS-CoV-2) infection has urged the investigation of preventive vaccines. Recently, our team has developed a recombinant protein vaccine, targeting receptor binding domain (RBD) of the spike protein (S-RBD) of SARS-CoV-2, which could induce a potent antibody response and protect non-human primates from SARS-CoV-2 challenge.^1^ The recombinant RBD protein is proved as a potent antigen and a novel adjuvant is in demand for the effective stimulation of adaptive immunity. Therefore, to improve the efficacy of the vaccine and seek a novel adjuvant that can stimulate both humoral and cellular immunity, we investigated the potential of series of cationic nanocarriers as adjuvants of the recombinant S-RBD vaccine for SARS-CoV-2. As the surface charge of a nanocarrier might dramatically affect the immunogenicity of a vaccine and enhance and/or shape antigen-specific immune responses, we also used anionic nanocarriers and neutral nanocarriers as controls (Supplementary Table S1). S-RBD vaccines with adjuvant candidates were administered intranasally or intramuscularly in the present study.

Three cationic nanocarriers, namely, polyethyleneimine (PEI), N-[1-(2,3-Dioleoyloxy) propyl]-N,N,N-trimethylammonium chloride (DOTAP) and Chitosan were investigated for their adjuvant effects. Anionic liposome (AnionicL) and neutral liposome (NeutralL) were used as control. AnionicL was composed of cholesteryl hemisuccinate, phosphatidylcholine (PC) and cholesterol, while NeutralL was simply composed of PC and cholesterol. The ELISA results showed that the RBD-specific IgM and IgG titers in the serum were higher when RBD vaccine was intranasally administered with cationic nanocarriers than that of RBD group or AnionicL/NeutralL group (Fig. 1a). The increase of total antibody levels reached a peak when animals were vaccinated using PEI as an adjuvant (Fig. 1a). Compared with AnionicL and NeutralL, cationic nanocarriers, especially PEI, significantly improved RBD vaccine-induced IgG1 antibody response (Th2-associated^2^), as well as IgG2a and IgG2b responses (Th1-associated^3^) (Fig. 1a). Similar findings were acquired in the intramuscular vaccination model (Fig. 1b). These data indicated that cationic nanocarriers enhanced RBD-specific humoral immunity by stimulating Th1- and Th2-associated immune responses through both intranasal and intramuscular vaccination.

**Fig. 1:**
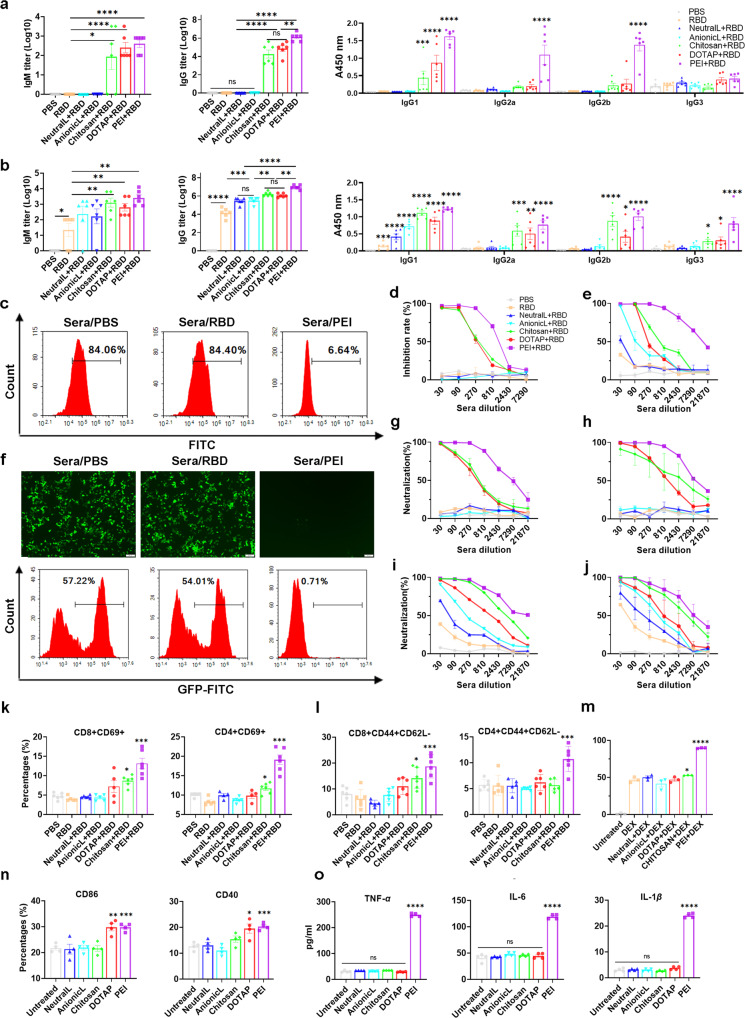
Cationic nanocarriers are potent adjuvants for recombinant S-RBD protein vaccine of SARS-CoV-2. **a, b** Cationic nanocarriers enhanced RBD-induced humoral immune response. NIH mice (*n* = 6/group) were immunized intranasally (**a**) and intramuscularly (**b**) with RBD+ nanocarriers (5 μg RBD/mouse). On day 14 after the first dose, immune sera were collected and titers of the total IgM were examined by ELISA. On day 35, total IgG levels and IgG isotypes were also determined. *p* value of IgG isotypes was compared to PBS group. **c–j** Sera were collected on day 35 for functional characterization. Blockade of RBD binding to cell surface ACE2 receptor by immune sera from intranasally (**c, d**) and intramuscularly (**e**) immunized mice was measured by flow cytometry (FCM). Neutralization of EGFP-expressing SARS-CoV-2 pseudovirus infection to 293T/ACE2 cells by immune sera from intranasally (**f**, **g**) and intramuscularly (**i**) immunized mice was measured with fluorescent microscopy and FCM. Scale bar, 100 μm. **c** and **f** were performed at 1:270 dilution. Neutralization of luciferase-expressing SARS-CoV-2 pseudovirus infection in 293T/ACE2 cells by immune sera from intranasally (**h**) and intramuscularly (**j**) immunized mice. *n* = 3. **k**, **l** Cationic nanocarriers + RBD vaccine induced cellular immunity. NIH mice were immunized as aforementioned. We isolated lymphocytes from inguinal lymph nodes and further analyzed the proportion of activated CD69^+^CD8^+^ and CD69^+^CD4^+^ T cells (**k**) and effector memory (CD44^+^CD62L^−^) T cells in CD4^+^ and CD8^+^ T cell populations (**l**) by FCM. *n* = 6. *p* was compared to PBS group. **m** After being separated and cultured for 7 days, DCs were cultured in medium with DEX or DEX+ nanocarriers. Then the uptake of DEX by DCs was analyzed by FCM. *n* = 3. *p* value was compared to DEX group. **n** DC were stimulated by nanocarriers for overnight. Maturation surface markers (CD86, CD40) were analyzed by FCM. **o** Inflammatory cytokines (IL-6, IL-1*β*, and TNF-*α*) in the supernatants were detected by ELISA. *n* = 4. *p* was compared with untreated group. All the data were presented as mean ± SEM. NS: not significant

For functional antibody characterization, immune sera from intranasally vaccinated mice were collected on day 35 to test its blocking activity of RBD to angiotensin converting enzyme II (ACE2) receptor. Immune sera from the mice vaccinated with phosphate buffered saline (PBS) or RBD alone had no inhibitory activity with the appearance of over 84% RBD-ACE2 binding in 293T/ACE2 cells (293T cells stably expressing ACE2) (Fig. 1c). In contrast, after being treated with sera from the mice immunized with PEI+RBD, only 6.64% 293T/ACE2 cells were detected positive (Fig. 1c). Immune sera from the mice vaccinated with cationic nanocarriers+RBD were more effective in blocking RBD-ACE2 specific binding, with 50% inhibiting titers of 1:810 (PEI) and 1:270 (DOTAP and Chitosan) compared to other groups (Fig. 1d). We also observed similar results in the intramuscularly vaccinated model (Fig. 1e). We next performed a neutralization assay to assess the neutralizing capability of immune sera against SARS-CoV-2 pseudovirus infection into 293T/ACE2 cells. Cells infected by SARS-CoV-2 pseudovirus were recorded as EGFP positive. We observed that the number of EGFP-expressing cells decreased sharply when cells were incubated with immune sera from mice intranasally immunized with PEI+RBD at 1:270 dilution, in comparison to that treated with immune sera from mice vaccinated with PBS or RBD alone (Fig. 1f). Sera from mice immunized with PEI + RBD, DOTAP + RBD, and Chitosan + RBD exhibited a neutralization rate of 99.01, 64.23, and 70.68% at the same dilution, respectively (Fig. 1g). In contrast, AnionicL and NeutralL failed to improve the production of protective antibodies induced by RBD vaccination in mice (Fig. 1g). These findings were further proved in a luciferase assay (Fig. 1h). The intramuscular immunization model also showed similar trends (Fig. 1i, j). In summary, these data suggested that cationic nanocarriers are able to increase the RBD-induced humoral immunity significantly, which was associated with the production of functional antibodies with a strong viral neutralizing activity.

T cells are closely concerned with the clearance of intracellular infected pathogens. In the next set of experiments, we subsequently evaluated whether cationic nanocarriers can improve the stimulation of cell-mediated immunity. Our results revealed that immunization of mice with PEI + RBD or Chitosan + RBD dramatically increased the proportion of CD69^+^CD4^+^ and CD69^+^CD8^+^T cells in the inguinal lymph nodes (Fig. 1k). The CD69 antigen is known as the earliest activation marker on the surfaces of antigen-specific activated lymphocytes.^4^ These results indicated that cationic nanocarriers helped enhance the activation of cytotoxic CD8^+^ T lymphocytes and CD4^+^ T helper arm. In addition, CD44 is a hallmark of memory T cells that can be further divided into central memory (CD44^+^CD62L^+^) and effector memory (CD44^+^CD62L^−^) T cells.^5^ We discovered that the percentages of effector memory T cells in both CD4^+^ and CD8^+^ T cell populations significantly increased in the PEI+RBD group while compared with control groups (Fig. 1l). Despite the slight increase found in the proportion of effector memory CD8^+^ T cells in Chitosan + RBD group (Fig. 1l), notably, in other groups, there was no significant increase in the percentages of activated and memory T cells in both CD4^+^ and CD8^+^ T cell populations in either intranasal or intramuscular vaccination model (data not shown). These observations indicated that cationic nanocarriers, especially PEI, may promote more potent antiviral effects via enhancing the activation of cellular immunity.

Efficient presentation of extracellular antigens and activation of dendritic cells (DCs) are essential for the initiation of immune responses. Next, we examined whether cationic nanocarriers influence the activation of DCs using FITC-labeled dextran (DEX). As expected, PEI significantly enhanced the antigen uptake capability of DCs in vitro (Fig. 1m). Chitosan also showed a slight influence, while other nanomaterials, including cationic DOTAP, showed no effects on the antigen uptake capability of DCs (Fig. 1n). PEI promoted the maturation of DCs (Fig. 1n). In addition, PEI increased the secretion of inflammatory cytokines from DCs which included tumor necrosis factor (TNF)-*α*, interleukin (IL)-1*β* and IL-6 (Fig. 1o).

For safety assessment, we monitored mice appearance, body weight, excretion of feces and urine, and performed pathological evaluation of the vital organs in immunized mice. No significant differences were observed between the mice immunized with different nanocarriers and normal mice either in intranasal or intramuscular vaccination model, suggesting the good safety profile of these cationic adjuvants.

In conclusion, we discovered that cationic nanocarriers were potent adjuvants for mucosal and intramuscular vaccination of the recombinant RBD vaccine of SARS-CoV-2. They not only improved the RBD-induced humoral immunity but also enhanced the cellular immune response in comparison to control groups. These effects may be partially related to the increased antigen uptake and activation of DCs. What’s more, immune sera from the mice vaccinated with RBD and cationic nanocarriers effectively blocked RBD binding to cell surface ACE2 receptor and neutralized SARS-CoV-2 pseudovirus infection in 293T/ACE2 cells in vitro. Cationic nanocarriers are safe, easy to prepare, of low cost and highly adaptable, and they can also be tailored specifically to fulfill the requirements of different vaccines. To sum up, our findings suggested the potential of cationic nanocarriers as novel adjuvants to enhance the immune responses of the broadly designed formulations of SARS-CoV-2 vaccines.

## Supplementary information

Cationic nanocarriers as potent adjuvants for recombinant S-RBD vaccine of SARS-CoV-2
