# Supplementary material for: Cationic nanocarriers as potent adjuvants for recombinant S-RBD vaccine of SARS-CoV-2
Source: Signal Transduct Target Ther. 2020 Dec 11;5:291. doi: 10.1038/s41392-020-00434-x (PMC7729145; doi:10.1038/s41392-020-00434-x)
Supplement: Supplementary file 1 — Cationic nanocarriers as potent adjuvants for recombinant S-RBD vaccine of SARS-CoV-2 [file 41392_2020_434_MOESM1_ESM.docx]

Supplementary Materials for

Cationic nanocarriers as potent adjuvants for recombinant S-RBD vaccine of SARS-CoV-2

Hong Lei^1,+^, Aqu Alu^1,+^, Jingyun Yang^1^, Cai He^1^, Weiqi Hong^1^, Zesheng Cheng^1^, Li Yang^1^, Jiong Li^1^, Zhenling Wang^1^, Wei Wang^1^, Guangwen Lu^1,2^, Xiawei Wei^1,*^

1. *Laboratory of Aging Research and Cancer Drug Target, State Key Laboratory of Biotherapy and Cancer Center, National Clinical Research Center for Geriatrics, West China Hospital, Sichuan University, Chengdu, 610041, China*
2. *Emergency Department, State Key Laboratory of Biotherapy, West China Hospital, Sichuan University, Sichuan, People's Republic of China*

+ These authors contributed equally to this work

* Correspondence: Xiawei Wei (xiaweiwei@scu.edu.cn)

**This file includes:**

Materials and Methods

Supplementary Text

Supplemental Tables S1 and S2

**Materials and Methods**

1. **Materials**

Recombinant RBD protein with Fc fragment (RBD-Fc) of SARS-CoV-2 was purchased from Sino Biological. SARS-CoV-2 S-RBD recombinant protein was prepared by our group as previously described.^1^ N-[1-(2,3-Dioleoyloxy) propyl]-N,N,N-trimethylammonium chloride (DOTAP), polyethyleneimine (PEI, 25k), chitosan powder (25-190kD, low molecular weight), cholesteryl hemisuccinate (HEMI), phosphatidylcholine (PC), cholesterol (Chol) were purchased from Sigma (USA). Goat anti-mouse IgG, IgG1, IgG2a, IgG2b and IgG3 peroxidase conjugates were purchased from Southern Biotech.

1. **Cells and animals**

293T cells that highly express ACE2 receptor (293T/ACE2) were generated in our laboratory as previously reported.^1^ Both 293T cells and 293T/ACE2 cells were maintained in Dulbecco’s modified Eagle’s medium (DMEM, Thermo Fisher Scientific, USA) with 10% fetal bovine serum, 0.1mg/mL Streptomycin and 100U Penicillin at 37 °C with 5% CO_2_.

Female NIH mice at 6-8 weeks were from Vital River (Beijing, China) and acclimatized for one week prior to use. All animals were raised at pathogen-free conditions and weighed twice per week. All animal experiments have been approved by the Institutional Animal Care and Use Committee of Sichuan University (Chengdu, Sichuan, China).

1. **Liposome production**

All liposomes were produced by conventional evaporation way. Appropriate amounts of each lipids were dissolved in chloroform in a round bottom flask and were evaporated to dryness and desiccated for 1 h under reduced pressure at 55 ℃. The film was resuspended in the phosphate buffered saline (PBS) and vortexed at room temperature for 30 min. Neutral liposomes (NeutralL) were composed of only PC and Chol. Anionic liposomes (AnionicL) consisted of anionic HEMI, PC and Chol. Cationic liposomes were prepared by adding DOTAP and Chol. Chitosan was dissolved in 1% (v/v) acetic acid with constant stirring and the pH was then raised to 4.6–4.8 with NaOH. PEI was directly dissolved in PBS. Zeta-potentials and sizes of all nanocarriers were examined by Malvern Nano-ZS 90 laser particle size analyzer (Table S1).

1. **Immunization and sampling schedule**

NIH mice were divided into seven groups (6 mice / group). Vaccinate animals intranasally and intramuscularly with PBS, RBD only, RBD+NeutralL, RBD+AnionicL, RBD+Chitosan, RBD+DOTAP and RBD+PEI. Each group of mice were immunized with 50 µL volume on Day 0 then boosted on day 7 and day 21 with identical doses (Table S2). To monitor the induction of serum antigen-specific antibodies, blood samples were collected on day 14, 21, 28 and 35 via the eye socket vein. Sera were kept at -20 °C before use.

1. **Measurement of SARS-CoV-2 RBD Specific antibodies**

RBD-specific serum antibodies (including IgM, IgG, IgG1, IgG2a, IgG2b and IgG3) were determined by enzyme-linked immunosorbent assay (ELISA). Coat 96-well NuncMaxiSorp plates (Thermo Scientific, USA) with 100 µL of 0.1 µg/mL RBD solution dissolved in carbonate buffer (pH 9.5) for 12 h at 4 °C. Wells were then washed for 3 times using PBS with 0.05% (v/v) Tween 20 (PBS/T) and blocked with 1% bovine serum albumin (BSA) for 1 h at 37 °C. Incubate the plates with 100 µL of a series of diluted sera samples for 1 h at 37 °C. After 3 washes, treat the plates with 100 µL of anti-goat IgM, anti-mouse IgG, IgG1, IgG2a, IgG2b and IgG3 horseradish peroxidase conjugates diluted 1:5000 with 1% BSA for 1 h at 37 °C. After washing for 5 times, add 100 µL of 3,3',5,5'-tetramethylbiphenyldiamine (TMB) were to each well. Then, incubate for 10 min and use 50 µL/well of 1.0 M H_2_SO_4_ stopping solution to stop the reaction. The optical density was measured at 450 nm (A450nm).

1. **Blockade of RBD-Fc binding to receptor ACE2**

Blockade of RBD-Fc binding to cell surface ACE2 receptor was carried out by flow cytometry (FCM). Briefly, 0.3 μg/mL of RBD-Fc was incubated in the absence or presence of immune sera at different dilutions for 1 h at 37 °C. Harvest 293T/ACE2 cells and wash with PBS twice. Add the incubated mixture to harvested cells (2*10^5^/tube) and further incubate for 30 min at 4 °C. Wash cells for 3 times with PBS and stain them with FITC labeled anti-human IgG Fc secondary antibody (Sigma-Aldrich, St. Louis, MO, USA) for 30 min at 4 °C. After that, the mean fluorescent intensity (MFI) of each group was measured by NovoCyte Flow Cytometer (ACEA Biosciences, Inc.). The results were analyzed by FlowJo software.

1. **Neutralization of pseudovirus infection**

The SARS-CoV-2 pseudovirus system was generated by our team in a previous study.^1^ Luciferase-expressing pseudotype viruses were produced by three plasmids, including plasmids encoding codon-optimized SARS-CoV-2 S protein (1 μg) and plasmid psPAX2 (3 μg), luciferase-expressing HIV-1 genome (pNL4-3.luc.RE 8 μg). To produce EGFP-expressing pseudovirus, replace luciferase-expressing HIV-1 genome with pLenti-EGFP vector. All plasmids with 45 μg transfection reagent PEI were added to 700 µL of opti-MEM. Incubate for 15 minutes at room temperature and co-culture with 293T cells for 6 h. Then replace the supernatant with fresh medium. Supernatants containing pseudoviruses were harvested at 48 h and 72 h post-transfection. The infectivity of SARS-CoV-2 pseudovirus was titrated and 100 TCID_50_ (50% tissue culture infectious dose) was used for infection of 293T/ACE2 cells.

Preincubate pseudovirus with serially diluted immune sera in 96-well plates for 1 h at 37 °C. Then add 2*10^4^ 293T/ACE2 cells/well and incubate for additional 48 h. The infection of EGFP-expressing pseudovirus in 293T/ACE2 cells was determined by fluorescent microscopy and FCM. To test the infection of luciferase-expressing pseudovirus, remove the supernatants of infected cells. Then add 50 µL PBS, 50 µL lysis reagent from a luciferase kit and luciferase substrate (Promega). Detect relative light units with the Ultra luminometer.

1. **Detection of activated and memory T cells in the lymph nodes**

35 days after the first immunization, the immunized mice were sacrificed and the inguinal lymph nodes were taken. Lymph node samples were minced using a pestle and passed through a 70 μm nylon mesh in 10 mL PBS. Then samples were centrifuged at 300 g for 5 min and the supernatants were discarded. T cells were analyzed by FCM using following antibodies: CD3(PerCP/Cyanine5.5), CD4(APC), CD8(FITC), CD44(Brilliant Violet 421™), CD62(Brilliant Violet 510™) and CD69(PE). Activated T cells are defined as CD3^+^ cells expressing CD69. Effector memory and central memory of helper (CD4^+^) or [cytotoxic](javascript:;) (CD8^+^) T lymphocytes are defined as T cells (CD3^+^) expressing CD44^+^CD62L^-^ and CD44^+^CD62L^+^, respectively.^2^

1. ***In vitro* antigen uptake assay of dendritic cells (DCs)**

Isolate DCs and add 5*10^5^ cells/well in 12-well plates. FITC-dextran (DEX) was used as a mock antigen to investigate the effect of nanocarriers on the phagocytic ability of DCs. 1 mg DEX was pre-incubated with 10 μg of the five nanocarriers respectively for 10 min. DEX alone or with nanocarriers were then co-incubated with DCs for 1 h at 37 °C. Cells were repeatedly washed for 3 times with PBS and the uptake of DEX was determined by FCM. For maturation, DCs were cultured in 12-well plates and incubated overnight with 5 *µ*g/mL of nanocarriers (NeutralL, AnionicL, DOTAP, Chitosan and PEI). Both stimulated and unstimulated DCs were stained with fluorochrome-conjugated antibodies against: CD11C (PE/Cyanine7), CD40 (APC), CD80 (FITC), CD86 (PE) and MHCII (APC/Cy7). Inflammatory cytokines (IL-6, IL-1*β* and TNF-*α*) in the [supernatant](javascript:;) were detected by ELISA.

1. **Pathological evaluation of vital organs**

Mice were sacrificed 35 days after the first immunization. Vital organs including lung, heart, liver, kidney and spleen were isolated and fixed at 4% buffered formalin for at least 24 h and embedded in paraffin. Then we cut them into 3 mm thick sections and performed haematoxylin-eosin (H&E) staining following the manufacturer’s instructions. Stained slices were scanned with an upright microscope (Nikon).

1. **Statistical Analysis**

We performed statistical analysis with GraphPad Prism 8. Statistical values were tested by a two-tailed unpaired Student’s t-test. Data were expressed as the mean ± SEM. P-values < 0.05 were considered statistically significant (*P < 0.05; **P < 0.01; ***P < 0.001; ****P < 0.0001).

**References**

1 Yang, J. *et al.* A vaccine targeting the RBD of the S protein of SARS-CoV-2 induces protective immunity. *Nature*. **586**, 572-577, (2020).

2 Roberts, A. D., Ely, K. H. & Woodland, D. L. Differential contributions of central and effector memory T cells to recall responses. *J Exp Med*. **202**, 123-133, (2005).

**Supplementary Text**

Abbreviations

SARS-CoV-2: severe acute respiratory syndrome coronavirus 2; RBD: receptor binding domain; S-RBD: RBD of the spike protein; DOTAP; N-[1-(2,3-Dioleoyloxy) propyl]-N,N,N-trimethylammonium chloride; PEI: polyethyleneimine; AnionicL: anionic liposome; NeutralL: neutral liposome; PC: phosphatidylcholine; ACE2: angiotensin converting enzyme II; DCs: dendritic cells; DEX: FITC-labeled dextran

**Table S1** Size and zeta-potential of nanocarriers

| Nanocarriers | Mean size (nm) | PDI | Zeta-potential |
| --- | --- | --- | --- |
| PEI | 15.6±0.38 | 0.46±0.02 | 10.0±1.06 |
| DOTAP | 72.0±4.3 | 0.13±0.08 | 41.9±1.77 |
| Chitosan | 227±46.9 | 0.46±0.08 | 19.8±0.35 |
| NeutralL | 155.3±0.95 | 0.28±0.003 | -2.865±0.13 |
| AnionicL | 126.1±0.2 | 0.25±0.003 | -26.7±1.76 |

Data were denoted as mean±SD.

**Table S2** Immunization formulas

| Group | Formula /μg per mouse  Adjuvant+RBD |
| --- | --- |
| PBS | - |
| RBD | 0+5 |
| PEI+RBD | 100+5 |
| DOTAP+RBD | 300+5 |
| Chitosan+RBD | 300+5 |
| NeutralL+RBD | 300+5 |
| AnionicL+RBD | 100+5 |
